# Ureterorenoscopic treatment of ureteral stones – influence of operator’s experience and skill on the procedure outcome

**DOI:** 10.3325/cmj.2011.52.55

**Published:** 2011-02

**Authors:** Davor Librenjak, Marijan Šitum, Dijana Gugić, Kazimir Milostić, Mario Duvnjak

**Affiliations:** 1 Department of Urology, Clinical Hospital Center Split, Split, Croatia; 2 Department of Pathology, Forensic Medicine and Cytology, Clinical Hospital Center Split, Split, Croatia

## Abstract

**Aim:**

To observe the influence of operating urologist’s education and adopted skills on the outcome of ureterorenoscopy treatment of ureteral stones.

**Methods:**

The study included 422 patients (234 men, 55.4%) who underwent ureterorenoscopy to treat ureteral stones at the Urology Department of Clinical Hospital Center Split, Croatia, between 2001 and 2009. All interventions were carried out with a semi-rigid Wolf ureteroscope and an electropneumatic generator used for lithotripsy. The operating specialists were divided into two groups. The first group included 4 urologists who had started learning and performing endoscopic procedures at the beginning of their specialization and the second group included 4 urologists who had started performing endoscopic procedures later in their careers, on average more than 5 years after specialization.

**Results:**

Radiology tests confirmed that 87% (208/238) of stones were completely removed from the distal ureter, 54% (66/123) from the middle ureter, and 46% (28/61) from the proximal ureter. The first group of urologists completed significantly more procedures successfully, especially for the stones in the distal (95% vs 74%; *P* = 0.001) and middle ureter (66% vs 38%; *P* = 0.002), and their patients spent less time in the hospital postoperatively.

**Conclusion:**

Urologists who started learning and performing endoscopic procedures at the beginning of their specialization are more successful in performing ureteroscopy. It is important that young specialists receive timely and systematic education and cooperate with more experienced colleagues.

Ureterorenoscopy was introduced to the clinical practice in the 1980s and since then it has become a widely accepted and reliable method for ureteral stone treatment with few complications (1,2). It is the method that fully meets the main principle of minimally invasive surgery – to achieve full recovery with minimal surgical trauma (1,3,4). Since it uses a smaller working-channel caliber, continuous irrigation, and application of video carts, ureterorenoscopy enables a more thorough exploration and optimal approach to all stones throughout the ureter’s full length.

The success rate (stone-free rate) of ureterorenoscopy in the proximal, middle, and distal ureter is around 80%, 90%, and 95%, respectively (1,5-7). Compared with shockwave lithotripsy (SWL), ureterorenoscopy has a higher stone-free rate for stones smaller than or equal to 10 mm in the distal ureter and stones bigger than 10 mm in the proximal ureter (1,2,5,8).

Besides the influence of stone position and size, the efficiency of the procedure depends on the experience and skill of the operating urologist. The skill of the operator is listed as a contributing factor for the ureterorenoscopy success rate in the European Association of Urology guidelines (1), yet not one of the reviewed studies has ever specifically compared the two. We hypothesized that operators who had received systematic and gradual education in endoscopy by an experienced mentor at the beginning of their urologic specialization were more successful in performing ureterorenoscopy than urologists who had started learning endoscopy later in their career. This study examined how education, skill, and experience of the operating urologist influenced the postoperative success rates in patients who underwent ureterorenoscopy.

## Patients and methods

This retrospective clinical study analyzed ureterorenoscopy results in 422 patients (234 men, 55.4%) at the Urology Department of Clinical Hospital Center in Split, Croatia, between 2001 and 2009. The indications for ureterorenoscopy included ureteral stones, relentless renal colics, obstructive uropathy with or without an infection, and stones in the proximal ureter that had not been removed before by SWL.

All procedures were performed by 8 urologists. The first group included 4 urologists who had been gradually and systematically educated in endoscopy since the beginning of their 4-year specialization. Their education started with the training of basic skills and advanced step by step to performing of transurethral surgery (transurethral resection of prostate, transurethral resection of bladder tumors, internal urethrotomy), either through a constant cooperation with an older colleague or through international exchange visits to similar urological clinics in the UK. The second group included 4 urologists who had started performing transurethral surgery procedures later in their careers, on average more than 5 years after their specialization. They were either self-taught, using trial and error method, or were loosely supervised by more experienced colleagues but lacked basic ureterorenoscopy training.

Both groups of operators were comparable considering their experience and duration of urological career. Before 2001, ureterorenoscopy procedures at Urologic Department of Clinical Hospital Split had been sporadically performed, mostly by the second group of operators (45 vs 5).

Most of the operating procedures were performed with spinal anesthesia with Lidocaine. General anesthesia, induced and maintained with Isoflurane, was used when the stones were located in the middle and proximal ureter. All interventions were carried out with a semi-rigid Wolf 10.5 Ch ureteroscope with a 4.8 Ch working channel width (Richard Wolf Gmbh, Knittlingen, Germany). An electropneumatic generator, Lithoclast 2290 Luxury Cart (Swiss Lithoclast, Nyon, Switzerland), was used for lithotripsy. Smaller stones were retrieved with tweezers or Dormia basket for stone extraction. Preoperative antibacterial prophylaxis (1 g of cephasolin, a single dose applied intravenously 2 hours before the procedure) was administered to all patients, after which they were given uroantiseptics for a week (second generation kinolons). In case of severe postoperative complications, patients were put on a prolonged antibiotics regimen.

Patients’ histories and postoperative findings and urologists’ autobiographical information were collected, as well as the data on stone position and size (measured with intravenous urography), lateralization in the body, number of calculi, patient’s sex, postoperative findings, and complications if present. To estimate the size of the ureteral stone we routinely took x-ray images of the urinary tract right before the procedure. To assess the effectiveness of the procedure, imaging was repeated on the first or second day after the first ureterorenoscopy, meaning that if the patient underwent a second ureterorenoscopy after the two day period, the secondary data were not taken into consideration. The procedure was defined as: 1) successful when the ureteral stone was either completely disintegrated or removed in whole, or 2) unsuccessful when one of the following occurred: the stone was unreachable (regardless the cause); during the procedure the stone migrated into the kidney without having previously been reduced in size; the stone was not completely disintegrated; the stone was still present on the place of the original site but in a reduced volume; or there was any migration of stone particles into the kidney. In unsuccessful cases, and when the stone was organic, we performed retrograde pyelography one day after the procedure. Therefore, the stone-free rate in this study was defined as complete stone removal confirmed with a native x-ray or RP, one or two days after the operation.

### Statistical analysis

Independent potential risk factors were compared with the dependent variable (the procedure success) using multiple logistical regression analysis, binary marking the unsuccessful cases with 0 and successful cases with 1 (SPSS, version 15, SPSS Inc., Chicago, IL; USA; Backward-Wald method). Numerical data were compared by *t* test, frequencies were presented by absolute frequencies (number and percentage), and the two groups of operators were compared by χ^2^ and Mann-Whitney test. Statistica 8.0 software package was used (Statsoft, Tulsa, OK, USA). Statistical significance level in all analyses was set at *P* < 0.05.

## Results

There was no significant correlation between the length of hospital stay and the patient’s sex, and the lateralization of the stone. A total number of 238 (56%) patients had a stone in the distal ureter, 123 (29%) in the middle ureter, and 61 (15%) in the proximal ureter. No significant correlation was found between the patient’s sex and the stone position (χ^2^ = 3.18, *P* = 0.204) (Table 1). The stone was completely removed in 302 (72%) patients, while partial or no definitive therapeutic effect was achieved in 120 (28%) of the patients. There was no significant correlation between the patient’s sex and the postoperative success rates (χ^2^ = 0.10, *P* = 0.751). The postoperative success rate of completely removed stones from the distal ureter was 87%, 54% from the middle ureter, and 46% from the proximal ureter. Significant correlation was found between successfully removed stones and their position (χ^2^ = 68.0; *P* < 0.001), while no significant correlation was found between the year when the patient was treated and the success of stone removal (χ^2^ = 10.38, *P* = 0.321).

**Table 1:** Characteristics of patients who underwent ureterorenoscopy to treat ureteral stones by two groups of operators*

Patient characteristics	Total	First group	Second group
Men/women ratio (No.)	234/188	148/116	86/72
Age in years (median, range)	53 (17-86)	52 (17-86)	53 (19-83)
No. of stones in the left/right ureter	220/202	150/114	70/88
Stone position (No., %):			
proximal ureter	61	46 (17)	15 (9)
middle ureter	123	68 (26)	55 (35)
distal ureter	238	150 (57)	88 (56)
Stone size in millimeters (mean ± standard deviation)		8.80 ± 2.37	7.35 ± 3.39

*The first group of operators was systematically educated in endoscopy since the beginning of their specialization. The second group of operators was self-taught or loosely supervised in endoscopy.

### Comparison of success rates between the two groups of operators

The first group of specialists operated on 264 patients (148 men, 56%) and the second group of specialists operated on 158 patients (86 men, 54.4%). The majority of ureteral stones were located in the distal ureter (n = 238). One hundred fifty (63%) of all distally located stones were treated by the first group of operators and 88 (37%) were treated by the second group of operators. The smallest number of stones was located in the proximal ureter (n = 61). Forty six (75%) were treated by the first group and 15 (25%) by the second group of operators. Concerning stone localizations, the first group of operators treated more stones in the proximal ureter, the second group treated more stones in the middle ureter, while both groups treated a similar number of stones in the distal ureter (χ^2^ = 7.1, *P* = 0.028) (Table 1). The first group of operators had a significantly higher overall success rate (χ^2^ *=* 24.22, *P* < 0.001). It also had a significantly higher rate for both the distal and middle ureter, but not for the proximal ureter (Table 2).

**Table 2:** Comparison of ureterorenoscopic procedure outcomes between the two groups of operators*

	First group (264 patients)	Second group (158 patients)	*P*
Duration of hospitalization after ureterorenoscopy (mean days ± SD)	2.02 ± 1.93	2.52 ± 2.46	0.027^†^
Stones completely removed from (No, %):			
distal ureter	143 (95)	65 (74)	<0.001^‡^
middle ureter	45 (66)	21 (38)	0.002^‡^
proximal ureter	23 (50)	5 (33)	0.261^‡^

*The first group of operators was systematically educated in endoscopy since the beginning of their specialization. The second group of operators was self-taught or loosely supervised in endoscopy. SD – standard deviation.

†Mann-Whitney test.

‡χ^2^ test.

There was a significant difference in mean stone size between the two groups of operators, measured before the operation with intravenous urography (8.80 ± 3.34 vs 7.35 ± 2.37 mm; *t* = 4.72; *P* < 0.001). The first group of operators successfully removed significantly larger stones than the second group (8.34 ± 3.07 vs 7.14 ± 1.56 mm; *t* = 7.14; *P* < 0.001). The first group of operators successfully removed significantly larger stones from the distal ureter (7.89 ± 2.95 vs 6.98 ± 1.98 mm; *t* = 2.26; *P* < 0.001), but there was no significant difference between the groups in the size of successfully removed stones (median, range) from the middle ureter (10 [7-15] mm vs 7 [5-11]) mm; z = 1.36; *P* = 0.173) and the proximal ureter (8 [5-20]mm vs, 7 [5-12] mm; z = 1.76, *P* = 0.076). Also there was a significant difference between the two groups in stone size (median, range) in unsuccessful procedures for the proximal ureter (10 [6-20] mm vs 7 [4-20] mm; z = 2.10, *P* = 0.034), and the middle ureter (10 [5-20] mm vs 7 [5-13] mm; z = 3.83, *P* < 0.001), but not for the distal ureter (9 [7-15] vs 7 [5-15]; z = 1.49, *P* = 0.134).

Patients treated by the first group of operators spent significantly less time in the hospital after the ureterorenoscopy procedure (Table 2). Complication rate was 4.5% (12/264) for the first group of operators and 6.3% (10/158) for the second group. In the first group, there were 3 cases of ureteral lesions, 7 cases of pyelonephritis, 1 case of epididymitis, and 1 case of pneumonia. In the second group, there were 4 cases of ureteral lesions, 5 cases of pyelonephritis, and 1 excessive bleeding. This parameter, therefore, did not significantly differ between the groups (χ^2^ = 0.63; *P* = 0.430). We did not observe a positive correlation between stone localization and the number of complications in either of the two groups of operators.

The first group of operators more frequently used endoprostheses, mostly ureteral catheters for 24 hours: in 152 out of 264 patients (57.5%) vs 35 out of 158 (22.1%) patients (χ^2^ = 40.62; *P* < 0.001).

The first group did not significantly improve their operation success rates during the studied years, while the second group improved its success rates, but only for the stones in the middle ureter (*P* = 0.010, data not shown).

Multiple logistical regression analysis of independent variables showed that the group of operators (risk ratio [RR], 0.3; 95% confidence interval [CI], 0.2-0.5, *P* < 0.001), stone position in the middle ureter (RR, 5.6; 95% CI, 3.2-9.7; *P* < 0.001), the proximal ureter (RR, 7.6; 95% CI, 3.9-15, *P* < 0.001), and the stone size (RR, 1.1; 95% CI, 1-1.2, *P* = 0.04) were significantly correlated with the procedure success (Table 3). Other independent variables like age, sex, stone lateralization, and the year when the procedure was carried out were not correlated with the success rate of the procedure.

**Table 3:** Independent variables affecting the success rate of the ureterorenoscopy procedure

Variables*	*P^†^*	Relative risk (95% confidence interval)
Group of operators	<0.001	0.295 (0.18- 0.49)
Middle ureter stone	<0.001	6.03 (3.50-10.39)
Proximal ureter stone	<0.001	8.36 (4.25-16.45)
Stone size	0.04	1.08 (1.01-1.17)

*Variables were not correlated with the success rate: age, sex, stone lateralization, and the year when the procedure was carried out.

†Logistical regression; Backward-Wald method.

Received: June 28, 2010

Accepted: January 24, 2011

## Discussion

Our study confirmed that the basic determinants of ureterorenoscopy success were ureteral stone position and the size and skills of the operating urologist adopted through education in endoscopy.

Numerous studies have shown that this method is most effective for stones in the distal ureter, but less effective in the proximal ureter (1,9). It is also most effective for stones up to 10 mm in size (1). Since the results of ureterorenoscopy largely depend on the operator’s skill, all new urology specialists have to receive adequate and continuous training and satisfactory and timely practice in the operating room, preferably guided by a mentor.

We have not found any studies that concluded anything specific on how skill, education, or experience in endoscopy affect the success rates in ureterorenoscopy procedures. Our study found significantly greater ureterorenoscopy success rates for middle and distal ureteral stones in the first group of urologists. The success rates for proximal ureteral stones were also greater in the first group, although this finding was not significant.

The success rates and postoperative results for distal ureteral stones in the first group of urologists are easily comparable with the literature, whereas the results for the middle and proximal ureter are not so easily comparable (5-7). This may be due to two reasons. One is the somewhat outdated equipment, but as both groups of operators performed with the same material and in the same financial circumstances, the only difference was their education and innate skill. The other possible reason is the difference in the length of the follow-up periods. Stone-free rate in the literature usually reflects patients’ status a month after the procedure, while we monitored the short-term impact of ureterorenoscopy (during 1-3 days of hospital screening), along with the influence of operator’s skill, which is the most important factor in immediate postoperative ureterorenoscopy outcome. In fact, the long-term final procedure outcome and stone-free rate depend less on the operator’s skill and more on the effectiveness of supportive therapy, medication (hydration, spasmolytics, and expulsive therapy), and patient’s cooperation; none of which was directly measured in our study. Similar results for short-term stone-free rates have been published by Butler et al in 2004 (10).

Postoperative hospitalization was significantly shorter for patients treated by the first group of urologists. This is perhaps the result of a one-day preventive ureteral catheter drainage more often used by the first group of operators. The effectiveness of routine drainage after ureterorenoscopy and SWL procedures and justification for its use are still debated (11,12). Nonetheless; we believe that a 24-hour catheterization is useful in the prevention of possible postoperative obstruction and/or renal colics due to severe mucosal edema, a leftover stone particle, or a blood cloth within the ureter. The literature readily confirms this algorithm (13-15). The studies in which routine postoperative drainage was considered unnecessary dealt mainly with double-J endoprostheses, which are usually removed 1-2 weeks after the procedure (16-18). Both groups of operators in our study used the double-J endoprosthesis in exceptional cases of complications, such as ureteral lesion or previous long-term obstruction uropathy. The incidence of major procedure-related complications for both groups of operators was similar regardless of the location of the stone. All ureteral lesions were treated with double-J endoprosthesis, while, only in one case, the physicians immediately switched to an open procedure.

According to the literature, the incidence of ureteral lesions as complications is between 3% and 7% (3,9,19,20). We observed ureteral lesions in 3 patients operated on by the first group of operators (3/264, 1.1%) and in 4 patients operated on by the second group (4/158, 2,5%). In the late 1980s, when instruments with wider working channels were used (eg, 12.5 Ch), complications, including ureteral ruptures, occurred more often (21). Then the perforation incidence was 31% for the proximal ureter and around 8% for the middle and distal ureter (9). Contrary to our results, Schuster et al in 2001 found a correlation between the experience of the operator and the number of complications (22).

The limitation of this study is the small number of urologists per group, but this was determined by the capacity of our department (“department with limited case load”) and the retrospective character of our study. The study would benefit from a randomized prospective design but we took into consideration the patient’s right to choose his or her operator. In the everyday practice, the patient would be highly reluctant to enter a study in which he or she may be operated on by a randomly chosen surgeon or a surgeon who is potentially less experienced or skillful. We are not aware of any published prospective randomized study on this subject, and we believe that the issue of trust between the patient and physician is an important reason to choose a retrospective design.

In conclusion, our study showed that the operators who had begun training and practicing endoscopy procedures early in their specialization were more successful when performing ureteroscopy. This may indicate that they can easily and readily follow and adopt new technologies and methods on the market today, combining them with their already existing knowledge. Therefore, urology specialists need timely and systematic education and increased cooperation between urological clinics and departments, as well as referral centers and early educational programs for ureterorenoscopy procedures.
